# Histological Quantification of Angiogenesis after Focal Cerebral Infarction: A Systematic Review

**DOI:** 10.1155/2013/853737

**Published:** 2013-12-30

**Authors:** Wai Yin Leung, Matthew B. Jensen

**Affiliations:** Department of Neurology, University of Wisconsin, 1685 Highland Avenue No. 7273, Madison, WI 53705-2281, USA

## Abstract

Ischemic stroke is a leading cause of disability, and current treatments to improve recovery are limited. Part of the natural recovery process after brain injury is angiogenesis. The formation of new blood vessels around the infarct appears to be important for restoration of adequate perfusion to allow for healing of brain tissue. Many potential restorative treatments may affect, and be affected by, angiogenesis, so accurate quantification of this outcome is needed. We performed a systematic review of histological methods to quantify angiogenesis after cerebral infarction. We found reports of the use of a variety of histological and general and immunostaining techniques in conjunction with a variety of analysis methods. We found no direct comparison studies and concluded that more research is needed to optimize the assessment of this important stroke outcome.

## 1. Introduction

Ischemic stroke is a leading cause of disability, with few effective treatments available to improve recovery. Most ischemic stroke patients experience some degree of spontaneous recovery, but the natural processes leading to this recovery are unclear [1]. The formation of new blood vessels around the infarcted brain tissue, termed *angiogenesis*, appears to be important in restoring adequate perfusion to these healing areas [2]. Many aspects of angiogenesis and its contribution to stroke recovery are still unclear, and many potential restorative treatments may affect, and be affected by, angiogenesis; therefore, accurate quantification of this important tissue outcome is needed. We sought to review the available evidence for histological methods of quantification of angiogenesis after cerebral infarction.

## 2. Methods

We searched PubMed in May 2012 with the search terms histolog* AND angiogenesis AND stroke OR cerebral infarct* and Google Scholar with the search terms histology, angiogenesis, stroke, and cerebral infarction. We included full-text articles in English published prior to May 2012 of unique experiments describing a histological method for quantification of angiogenesis after cerebral infarction. We excluded abstracts and articles citing methods found in other articles if no modifications were described. Titles, abstracts, or full articles were reviewed to determine if each search result matched our selection criteria. We also reviewed the references of the selected articles for additional matching articles.

## 3. Results

The PubMed search returned 453 results, of which two met our selection criteria; we found one additional matching article in the references of the selected articles. The Google Scholar search returned 2000 results, of which six met our selection criteria; we found eight additional matching articles in the references of the selected articles. Due to overlap of the two searches, the total number of articles meeting our selection criteria was 10. We found variability in the species evaluated; the time interval between cerebral infarction and analysis; the thickness, interval, and number of brain sections analyzed; the number, anatomic location, and magnification of areas analyzed per brain section; and the specifics of the analysis method.

In 1994, Krupinski et al. reported histology of human brain tissue 5 to 92 days after focal cerebral infarction [3]. The authors stated that “infarcted areas and representative control tissues from the contralateral uninvolved brain hemisphere were collected,” without specifying anatomic locations. An unclear interval and number of 8 *μ*m thick sections were stained with hematoxylin and eosin “or for hemoglobin after Pickworth.” A variable number of areas on the sections were evaluated, “determined by the size of the tissue section and extent of the stroke damage.” Anatomic locations were not stated, but areas were evaluated “in each area of diseased and normal brains” at 400x magnification. A microvessel was counted if its profile was separated from other profiles. A total of 6520 microvessels of approximately 200 *μ*m or less in diameter were counted in 10,801 areas, with data presented as number per area.

In 1999, Bernaudin et al. reported histology of mouse brain tissue 1, 3, and 7 days after cerebral infarction [4]. An unstated number and interval of 20 *μ*m sections were stained for Epo and Epo-R and also for endothelial cells and microglia labeled with isolectin B4. “Immunopositive cells for Epo and Epo-R were identified by comparing the cellular morphology” based on the progression of infarct and specific immunomarkers such as isolectin B4. The number of areas analyzed was not stated, and images show that regions analyzed include different neuronal compartments and were done at “×250” and “×1000” magnification.

In 2001, Wei et al. reported histology of rat brain tissue at various time points for GLUT-1 and integrin *α*v*β*3 expression after cerebral infarction [5]. Some rats were injected with single doses of BrdU at 2, 4, 6, or 9 days after placing ligatures and then sacrificed 24 hours later. An unstated number of 14 *μ*m sections and every 5 sections were stained for GLUT-1. GLUT-1 was a marker for capillaries and any “colocalized BrdU indicates … angiogenesis.” And “labeled [BrdU] nuclei in the 2 vessels closest to each side of the core/infarct were counted in 4 or 5 sections through the stroke for each animal,” without further analysis details provided. Other rats were sacrificed at 3, 5, 7, or 10 days after ligations and an unstated interval and number of 14 *μ*m sections were stained for the endothelial cell marker integrin *α*v*β*3. Images of integrin *α*v*β*3 immunostained cells were shown using “higher power image of the field” in the “ischemic border and normal unstained cortex” without specifying the magnification and number of areas analyzed.

In 2003, Chen et al. reported histology of rat brain tissue 4 or 14 days after focal cerebral infarction, quantifying many aspects of angiogenesis [6]. Most of the rats had bromodeoxyuridine (BrdU) injections on each of the 14 days after cerebral infarction and fluorescein isothiocyanate (FITC) dextran intravenous injection prior to brain removal. For the FITC dextran analysis, 7 sections, 100 *μ*m thick, at 2 mm intervals from bregma 5.2 mm to −8.8 mm were prepared. Eight areas were imaged with a confocal microscope in the ipsilateral and contralateral hemispheres. The areas were selected on a reference section at bregma 0.8 mm; an illustration shows the anatomic locations relative to the infarct core, with 4 in the cortex and 4 in the striatum. “These regions were scanned in 512 × 512 pixel (279 × 279 *μ*m^2^) format in the *x*-*y* direction using a 4x frame-scan average, and 25 optical sections along the *z*-axis with a 1 *μ*m step-size were acquired under a 40x objective. Vascular branch points, segment lengths, and diameters were measured in three dimensions using software developed in our laboratory." FITC dextran data was presented for mean vessel diameter and length, branch point density per mm^3^, and total vessel surface area in *μ*m^2^ per mm^3^. Five 100 *μ*m coronal sections of unstated location and interval were immunostained for VEGF. The same eight areas were imaged as for the FITC dextran analysis, but at 20x magnification with a light microscope, and analysed with MCID image analysis system. “All values were presented as a percentage of the maximum value obtained,” without further details. BrdU immunostaining was performed on an unstated number and interval of 6 *μ*m sections. Endothelial cells with and without BrdU labeling in “10 enlarged vessels adjacent to the ischemic lesion were counted from each rat.” The same was done in “the contralateral homologous area,” without further details. “Vascular perimeter” data was also presented with these results, without further details. Immunostaining for VEGF receptors 1 and 2 (with flt-1 and flk-1 antibodies) was performed on an unstated number, interval, and location of 20 *μ*m thick sections, and “numbers of endothelial cells and numbers of flt-1 and flk-1 immunoreactive endothelial cells in 10 vessels adjacent to the ischemic lesion were counted from each rat,” without further details.

In 2007, Li et al. reported histology of mouse brain tissue 3, 7, 13, and 21 days after focal cerebral infarction [7]. BrdU was injected into animals once daily until day of sacrifice, and an unstated number of 10 *μ*m sections were stained for GLUT-1 as well as BrdU for double-label counting. “Every ninth section across the entire region of interest was counted” and 6 fields per section in the penumbra region were randomly chosen under 20x magnification of a light microscope and “repeated in 4 separate sections per brain.” Any morphological vessel-like structures stained with GLUT-1 and colocalized with BrdU would be counted as one immunopositive vessel on a confocal microscope. The ratio of BrdU vessels over total vessels counted is the quantification measure used for comparison between brain sections. Immunostaining for erythropoietin receptor (EpoR), a cytokine for red blood cell precursor, was also performed. Recombinant human erythropoietin was injected into the rats 30 minutes before the infarction and then once daily until day of sacrifice. The same vessel profile analysis technique used for BrdU/GLUT-1 was used for EpoR, and the “erythropoietin receptor expression in microvessels was detected by double immunostaining of anti-EPOR antibody and CD31 in the penumbra.” Erythropoietin receptor expression was measured by counting the number of EpoR-positive vessels on a confocal microscope and then calculating the ratio of this expression over the total number of vessels.

In 2009, Liao et al. reported histology of rat brain tissue 13 days after focal cerebral infarction [8]. A single 5 mm thick section “from bregma” was immunostained for VEGF and a marker or endothelial cells (CD31). Five “random” areas “around the infarction” were imaged at 200x magnification. “The number of CD-31 positive vessels was counted” without further details provided. Undefined “positive units” of VEGF were “computed,” presumably with unstated software, averaged from the 5 imaged areas, and a complex formula was used to “exclude the impact of nonspecific background signal.”

In 2010, Hu et al. reported histology of rat brain tissue 3, 7, or 14 days after focal cerebral infarction, including rats that were given physical exercises for the purpose of examining the link between physical exercise and angiogenesis [9]. An unstated number and interval of 20 *μ*m sections were stained for antiplatelet endothelial cell adhesion molecule-1 (PECAM-1), also known as cluster of differentiation 31 (CD31) to detect neogenetic blood vessels. The CD31 expression was quantified by averaging the total number of blood vessels on a light microscope at ×200 magnification field (×20 objective and ×10 ocular, 0.739 mm^2^ per field), “in 15 different microscopic fields per animal around the marginal zone of infarct region throughout brain sections.” Only one major criterion was provided for counting a blood vessel according to the Weidner's method, which is “any [CD31-stained] endothelial cell or endothelial cell cluster, clearly separate from adjacent microvessels, tumor cells, and other connective-tissue elements, was considered a single, countable microvessel” [10]. The average number of blood vessels counted in all 15 fields per animal provides a microvascular density (MVD), with which the experimenters “had further assessed” without other analysis details.

In 2011, Otilia et al. reported histology of human brain tissue 12 hours to 7 days after focal cerebral infarction, as well as for control cases [11]. An unstated number and interval of 5 *μ*m thick sections were immunostained for the angiogenesis promoter vascular endothelial growth factor (VEGF) and a marker of proliferative endothelial cells (CD105). An unstated number of images were taken at 60x magnification from unstated anatomic locations of the lesion, the contralateral hemisphere, and control brains. For analysis, “VEGF expression intensity was quantified as the sum of the integrated optical densities (IOD) of the threshold pixels for all signal measured in each image” using Image ProPlus 7 AMS software. The thresholding method was not stated. VEGF colocalization with CD105 was determined with “the Mander's M1 and M2 coefficients, which show the fraction of the intensity in each channel that coincide with some intensity in the other channel.”

In 2011, Li et al. reported histology of mouse brain tissue 14 or 30 days after focal cerebral infarction, including those given whisker stimulation [12]. All animals had injections of BrdU daily beginning on day 3 after ischemia until the day of sacrifice. An unstated number and interval of 14 *μ*m sections were stained for a few different primary antibodies which include anti-Glut-1 and anti-BrdU. “For systematic random sampling in design-based stereological cell counting,” 3 coronal brain sections spaced 276 *μ*m apart across the region of interest (from bregma +0.5 to bregma −0.5 mm) per animal were chosen. And then “4 fields per brain section were randomly chosen in the ischemic border region under 20x magnification of a light microscope or in confocal images.” Angiogenesis was quantified by “counting the number of Glut-1 positive vessels co-stained with BrdU” in the ischemic border region. Any morphological vessel-like structures stained with the positive endothelial cell marker are counted as a blood vessel. Results were analyzed by “the total cell counts in the 4 survey fields per brain section × 3 brain sections,” and some images were visualized three dimensionally to show “colocalization of Glut-1 and BrdU in the same cells.”

We found no reports of direct comparison studies of these or other methods.

## 4. Discussion

Accurate histological quantification of angiogenesis after cerebral infarction is necessary for a reliable assessment of the interaction of this tissue response to restorative treatments. Our review found reports of multiple histological techniques to quantify postinfarction angiogenesis in brain tissue, but there were no direct comparison studies. Most of the articles matching our selection criteria contained a method description that presented insufficient details for replication. In the absence of direct comparison data, some statements can be made regarding the relative strengths and weaknesses of the reported methods.

A general tissue stain such as hematoxylin-eosin staining (H&E) is both simple and widely used. It gives an overview of the structure of the tissue, enabling differentiation of the structures being examined as normal or pathological. However, this is more applicable in the case of identifying pathological or degenerated blood vessels rather than proliferation or new formation of blood vessels. This stain, overall, is economical and offers reproducible results.

Immunostaining for the vascular endothelial growth factor (VEGF) antibody was repeatedly described. This is mostly due to the well-established role of VEGF as a signal protein produced by cells that stimulate angiogenesis after cerebral ischemia. It also has been associated with neuroprotection and improved histological and functional outcomes from stroke through multiple mechanisms [13]. Therefore, this strategy makes sense for assessing the new formation of blood vessels in postischemic brains. It is, however, also important to note that overexpression of VEGF can be indicative of pathology [14].

CD31 is a transmembrane glycoprotein also designated as PECAM-1 (platelet endothelial cell adhesion molecule 1). It is present on the surface of platelets, monocytes, macrophages, and neutrophils and is a constituent of the endothelial intercellular junction. It plays a major role in the adhesion cascade between endothelial cells during angiogenesis and thereby qualifying it as an endothelial cell marker. It also facilitates leukocyte migration which is closely associated with the anti-inflammatory response exerted by a stroke onset; this further reinforces its role as an endothelial cell marker [15]. The advantage of CD31 can also become its potential weakness since its effect is mediated also by the brain's natural response to inflammation after cerebral infarction, and distinguishing between such effect and its induction of the angiogenic process can be difficult.

Bromodeoxyuridine (BrdU) is an exogeneous molecule that is taken up by dividing cells during DNA synthesis. Once incorporated into the new DNA, BrdU will remain in place and be passed down to daughter cells following division, which is why it is advantageous for labeling recently divided cells. And GLUT-1, a vessel marker, is often used in conjunction with BrdU. Double staining of BrdU and any vessel-specific marker such as GLUT-1 antibody would serve as a good approach to distinguish new vessels from old vessels already existing prior to ischemic infarction.

Integrin *α*v*β*3 is a heterodimer, glycoprotein adhesion molecule found on many cell types, including endothelial cells. Ligation of integrin *α*v*β*3 has been shown to be required for the survival and maturation of newly formed blood vessels, suggesting it to be an extremely important, or even necessary, angiogenic marker [16].

Erythropoietin (EPO) and erythropoietin receptor (EPOR) system demonstrated to have a key role during central nervous system embryogenesis, have increased expression during cerebral ischemia, and are still expressed in adult brain. After cerebral ischemia, EPO increase was delayed which is due to de novo synthesis of EPO [17]. EPOR is expressed by microvessels and neurons; in fact, an exogenous dose of erythropoietin is found to reduce infarct area through promoting angiogenesis [18]. This increases the reliability of using anti-EPOR antibody as an angiogenic marker.

Isolectin B4 is used to examine the microvessel diameter, and the lectin binds to an extracellular site on endothelial cells of brain blood vessels and thus can be used to label vessels in both living and paraformaldehyde-fixed slices. A potential weakness of isolectin B4 stain is that it is not specific just for endothelial cells, as it also binds to microglia.

To our knowledge, this is the first systematic review of this topic, but our review also has limitations. These methods are usually discussed in the context of larger overall studies designed to answer other questions, and therefore many studies using novel methods may not be indexed in a manner amenable to database searching. Additional applicable methods likely exist outside of the applied search terms as detailed in the methods. However, our search strategy was extensive and replicable and can be further expanded upon in the future to incorporate newly described methods. Additionally, reviewing the references of the selected articles allowed us to catch additional methods papers that are related to the topic.

In the absence of any direct comparative studies on different histological methods for quantifying angiogenesis, it is recommended that studies in the future perform the above histological methods with a systematic image analysis method all in a single study. Some of the studies presented did not provide details for image analysis methods, those that did used different analysis methods and are discussed to great length in this article. VEGF is a well-established endothelial cell marker, and combination of GLUT-1/BrdU produced excellent images with high signal-to-noise ratio in the studies presented in this paper hence; these may present as the more superior methods of assessing postcerebral infarction angiogenic processes. It is important to keep in mind that some of the studies discussed in this paper did not take into account the potential upregulation of angiogenesis due to experimental treatments such as stem cell transplantation, which can become a confounding variable when conducting a research study that directly examines the extent of angiogenesis using different histological methods. Other factors can also be of concern and invalidate the results by affecting the extent of angiogenic processes occurrence, such as variation in stroke volume, differences in anatomic location of interest for analysis, and timing of infarction and perfusion.

## 5. Conclusion

We conclude that the reported methods all seem reasonable, the technique descriptions have often been insufficiently detailed for replication, and the lack of comparative data makes statements about the superiority of any particular method impossible. Further research is needed to optimize the analysis of this important experimental outcome. We recommend studies directly comparing multiple modalities of assessment for angiogenesis to determine the most powerful method currently available.
